# Freiburg Neuropathology Case Conference: Posterior fossa tumour 15 years after microsurgical resection of a cerebellar pilocytic astrocytoma

**DOI:** 10.1007/s00062-024-01468-2

**Published:** 2024-10-23

**Authors:** M. Schwabenland, L. Becker, C. J. Gizaw, M. Prinz, H. Urbach, D. Erny, C. A. Taschner

**Affiliations:** 1 Departments of Neuropathology, University of Freiburg, Freiburg, Germany; 2 Department of Neuroradiology, Medical Center, University of Freiburg, Breisacherstraße 64, 79106 Freiburg, Germany; 3 Neurosurgery, University of Freiburg, Freiburg, Germany; 4 Medical Centre—University of Freiburg, Faculty of Medicine, University of Freiburg, Freiburg, Germany

**Keywords:** Pilocytic astrocytoma, Glioblastoma multiforme, Cerebellar metastasis, Primary central nervous system lymphoma, High-Grade astrocytoma with piloid features

## Case Report

A 52-year-old male patient was undergoing regular follow-up after surgical resection of a pilocytic astrocytoma (CNS WHO grade 1) located in the cerebellar vermis. In 2009, the patient underwent complete removal of the cystic tumour via a median suboccipital osteoplastic craniotomy at an external hospital. Postoperatively, his gait disturbance resolved completely over time. Annual magnetic resonance imaging (MRI) scans through 2022 showed no signs of residual tumour or recurrence, allowing for an extension of the follow-up interval to two years.

At the most recent follow-up, imaging revealed a contrast-enhancing cerebellar mass suspicious for tumour recurrence, although the patient remained asymptomatic. After discussion in the interdisciplinary brain tumour board, surgical treatment was recommended.

Microsurgical tumour resection was performed under general anesthesia, with the patient in the prone position, through a median suboccipital osteoplastic re-craniotomy. Intraoperatively, the tumour appeared orange-reddish, firm, and highly vascularized, resembling the macroscopic features of a hemangioblastoma. Numerous small tumour-supplying vessels in the surrounding parenchyma were identified and carefully severed before en bloc tumour removal was achieved. Intraoperative frozen section analysis suggested a glial-origin tumour. A thin layer of gray-altered glial tissue was also resected from the margins until they appeared free of visible tumour.

Postoperatively, the patient developed ataxia and dysarthria. His condition was further complicated by malabsorptive hydrocephalus, leading to psychomotor retardation and confusion. After the insertion of a ventriculoperitoneal shunt, the patient’s neurological condition gradually improved. Based on the histological findings, the patient was subsequently referred for adjuvant radiochemotherapy.

## Imaging

**Fig. 1:**
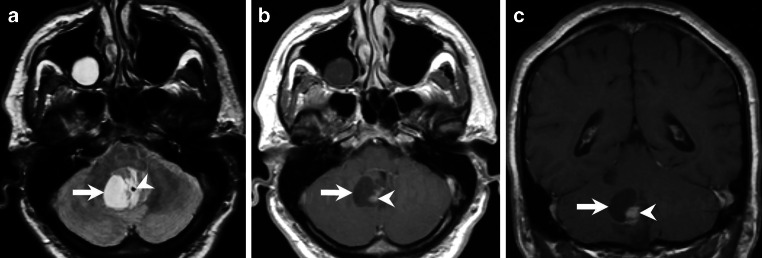
Preoperative magnetic resonance imaging (MRI) from 2009. Axial FLAIR (Fluid Attenuated Inversion Recovery) images (**a**) and coronal T2-weighted images (**b**) reveal a posterior fossa tumour with characteristic features of a pilocytic astrocytoma (**a**,**b**, *arrowhead*). On T1 weighted images obtained following intravenous administration of gadolinium (Gd) the solid component of the lesion showed homogeneous contrast enhancement (**c**, *arrowhead*). The solid tumour was surrounded by a large cystic portion (**a**–**c**, *arrow*). Neuropathological examination confirmed the diagnosis of pilocytic astrocytoma after microsurgical removal of the tumour in 2009

Initial magnetic resonance imaging (MRI) from 2009, obtained prior to the first microsurgical resection of a posterior fossa tumour, revealed a lesion with characteristic features of a pilocytic astrocytoma (Fig. 1a–c, arrowhead). The lesion showed homogeneous contrast enhancement following intravenous administration of gadolinium (Gd) on T1-weighted images (Fig. 1c, arrowhead) and included a large cystic component (Fig. 1a–c, arrow). Neuropathological examination confirmed the diagnosis of pilocytic astrocytoma after complete tumour removal was obtained in 2009.

**Fig. 2:**
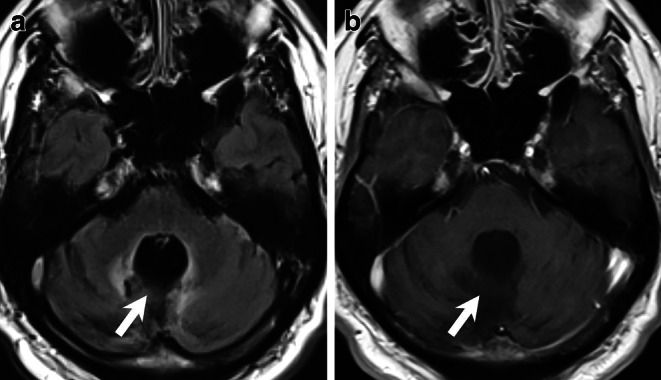
Bi-annual MRI follow-up from 2022. Axial FLAIR images (**a**), and axial T1-weighted images acquired after Gd administration consistently confirmed the complete removal of the tumour, with no evidence of recurrence at the site of the resection (**a**,**b**, *arrow*)

**Fig. 3:**
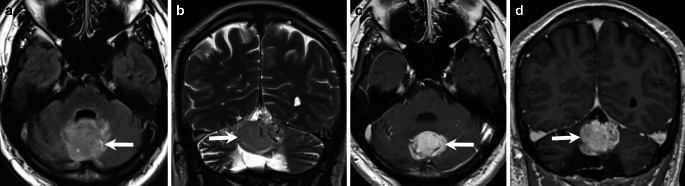
Bi-annual MRI follow-up from 2024 in the asymptomatic patient. On axial FLAIR images (**a**) and coronal T2-weighted images (**b**), the resection defect was filled with a lesion that appeared hyperintense compared to the surrounding cerebellar parenchyma (**a**,**b**, *arrow*). On axial (**c**), and coronal (**d**) T1-weighted images post-Gd administration, the lesion exhibited homogeneous contrast enhancement (**c**,**d**, *arrow*)

Up until 2022 (Fig. 2), annual MRI follow-ups consistently confirmed the complete removal of the tumour, with no evidence of recurrence at the site of the resection (Fig. 2, arrow). However, on MRI obtained in 2024 in the asymptomatic patient, the resection defect was filled with a lesion that appeared hyperintense on FLAIR and T2-weighted images compared to the surrounding cerebellar parenchyma (Fig. 3a,b, arrow). On T1-weighted images post-Gd administration, the lesion exhibited homogeneous contrast enhancement (Fig. 3c,d, arrow). Diffusion-weighted images demonstrated moderate diffusion restriction within the lesion (not shown).

## Differential Diagnosis

Lesions in the posterior fossa frequently present with overlapping clinical symptoms, including headaches, dizziness, and hearing impairment [1]. When these lesions compress the fourth ventricle, they can cause obstructive hydrocephalus, leading to signs of increased intracranial pressure, such as nausea, vomiting, and altered consciousness [1]. This region is particularly prone to tumour development in paediatric populations, where posterior fossa tumours are among the most common intracranial neoplasms [1]. In addition to tumours, a variety of other pathologies can affect the posterior fossa, such as vascular abnormalities (e.g., hemorrhage or malformations), congenital cysts, or infections like abscesses [1].

### Pilocytic Astrocytoma

Pilocytic astrocytomas are primary low-grade (Grade I) tumours of the central nervous system (CNS) that originate from astrocytes. These tumours are most commonly seen in younger patients and typically arise in the posterior fossa, often presenting with a large cystic component and a smaller mural nodule [2]. The absence of symptoms does not exclude tumour recurrence but may indicate slower progression compared to more aggressive forms of the disease [2–4].

In adults, pilocytic astrocytomas can occur in the cerebellum, though they are less common and may follow a different clinical course than in paediatric cases [5]. These tumours display a range of appearances on imaging: a large cystic component with a brightly enhancing mural nodule is present in 67% of cases, a non-enhancing cyst wall is seen in 21%, and an enhancing cyst wall in 46%, and 16% show a heterogeneous appearance with mixed solid components, multiple cysts, or central necrosis. About 17% of pilocytic astrocytomas appear completely solid on imaging. Tumour enhancement is almost universally observed (~95%), and up to 20% of cases may exhibit some degree of calcification. Hemorrhage is a rare complication [6].

### Cerebellar Glioblastoma multiforme

Glioblastoma multiforme (GBM) is the most aggressive type of primary brain tumours and is classified as a WHO Grade IV astrocytoma. While GBMs can occur in the posterior fossa, this is extremely rare, accounting for less than 1% of all GBMs [7]. On MRI, cerebellar GBMs typically present as heterogeneously enhancing lesions with central necrosis and significant perilesional edema, resembling the imaging characteristics of supratentorial GBMs. Key imaging features include necrotic cores, irregular borders, and surrounding vasogenic edema. Ring enhancement after gadolinium contrast is frequently observed, while diffusion-weighted imaging (DWI) often shows restricted diffusion due to tumour hypercellularity [3, 8].

Cerebellar GBMs can cause symptoms related to increased intracranial pressure, such as headaches, nausea, vomiting, and ataxia, due to their infratentorial location. Obstruction of cerebrospinal fluid flow can lead to hydrocephalus. Additionally, neurological symptoms may include dysmetria, vertigo, and gait disturbances, stemming from the tumour’s direct impact on cerebellar function [3, 9]. These tumours are highly proliferative, as indicated by elevated Ki-67 labeling indices, and carry a poor prognosis. Despite aggressive treatment involving surgical resection, radiation therapy, and chemotherapy, the median survival remains only 12–15 months [10, 11].

In our patient, a cerebellar GBM had to be considered in the differential diagnosis due to the presence of a large cerebellar mass. However, the limited presence of central necrosis and perifocal edema made GBM a less likely diagnosis in this case.

### Brain Metastasis

Brain metastases should be considered in the differential diagnosis, as they are the most frequently encountered malignant tumours in the central nervous system (CNS) in adults. The most common primary cancers that metastasize to the brain include lung cancer, breast cancer, and melanoma [12–14]. These metastatic lesions typically localize in juxtacortical areas and border zones within the brain parenchyma [15]. Although only 15% of brain metastases occur in the cerebellum, they remain the most frequently diagnosed malignant lesions in the posterior fossa [15, 16].

On MRI, brain metastases are generally isointense to hypointense on T1-weighted sequences and exhibit variable T2 signal intensity, depending on the histological characteristics and cellular composition of the metastasis [17]. Larger lesions often show ring enhancement after contrast administration, a feature commonly associated with necrosis and disruption of the blood-brain barrier [17]. Diffusion-weighted imaging (DWI) findings can vary based on the histology and cellular density of the tumour [18].

While CT is less effective in detecting smaller brain metastases, as they frequently appear isodense to brain parenchyma, it remains crucial for initial screening and identification of associated brain edema or hemorrhage [15, 17]. Hemorrhagic components can further complicate both CT and MRI appearances, leading to a range of radiological presentations [17]. Brain metastases are an important consideration in the differential diagnosis for this case, particularly given the cerebellar location, even in the absence of a known primary tumour [19, 20].

### Primary Central Nervous System Lymphoma

Primary central nervous system lymphoma (PCNSL) is a rare but aggressive form of non-Hodgkin lymphoma that affects the brain, spinal cord, or leptomeninges. The most common subtype is diffuse large B‑cell lymphoma (DLBCL) [21]. PCNSL typically presents as single or multiple enhancing lesions, most often in the supratentorial compartment, although they can also occur in the cerebellum [21, 22].

On magnetic resonance imaging (MRI), CNS lymphoma lesions are usually isointense to hypointense on T1-weighted images and hyperintense on T2-weighted images, with homogeneous contrast enhancement. These lesions often exhibit restricted diffusion on diffusion-weighted imaging (DWI), reflecting their high cellularity. Unlike gliomas or metastases, which more commonly show ring enhancement due to necrosis, CNS lymphoma typically presents with patchy to solid enhancement and limited central necrosis [23]. Perifocal edema is common but tends to be less pronounced than in cases of brain metastases or gliomas.

On CT, CNS lymphoma lesions frequently appear hyperdense because of their dense cellular structure [22]. An important diagnostic challenge arises when corticosteroids are administered, as steroid treatment can lead to a rapid and significant reduction in lesion size, potentially complicating the diagnosis if imaging is performed after steroid use [21, 24]. Given the cerebellar location and imaging features in this case, CNS lymphoma should be considered as a possible differential diagnosis.

### High-Grade Astrocytoma with Piloid Features

High-Grade Astrocytoma with Piloid Features (HGAP) is a rare but increasingly recognized entity, representing an aggressive variant of astrocytomas that shares histopathological similarities with pilocytic astrocytomas (PA) but exhibits features of high-grade malignancy. These tumours are classified as WHO Grade III or higher and display histological characteristics such as hypercellularity, nuclear pleomorphism, brisk mitotic activity, and microvascular proliferation [3, 25]. There is currently no consensus on specific imaging features, as HGAP tumours are heterogeneous in both location and appearance [26, 27]. Descriptions in the literature often depict these lesions as heterogeneously or peripherally enhancing masses with low T1 and high T2/FLAIR signal intensity. They may lack diffusion restriction in both central and peripheral components, with cystic areas, necrosis, and elevated perfusion also being reported. Additionally, there appears to be an association between HGAP and neurofibromatosis type 1 [26, 27].

Unlike low-grade PAs, HGAP tends to demonstrate rapid progression and recurrence, even after prolonged periods of stable disease following resection of a low-grade WHO tumour [28]. Molecular profiling is crucial for differentiating HGAP from other high-grade tumours like cerebellar glioblastoma, as distinction based on imaging alone is often difficult [25]. Clinically, HGAP is associated with a more aggressive course compared to classic PA, and symptoms such as headaches, ataxia, and neurological deficits may be prominent, particularly when the tumour is located in the cerebellum.

Despite aggressive treatments including surgical resection, radiotherapy, and chemotherapy, the prognosis for HGAP remains poor, with a median survival period shorter than that of lower-grade astrocytomas [15, 29]. Given the patient’s history of pilocytic astrocytoma and the current imaging findings, HGAP is a plausible diagnosis in this case.

## Histology, Immunohistochemistry and Molecular Analyses

**Fig. 4:**
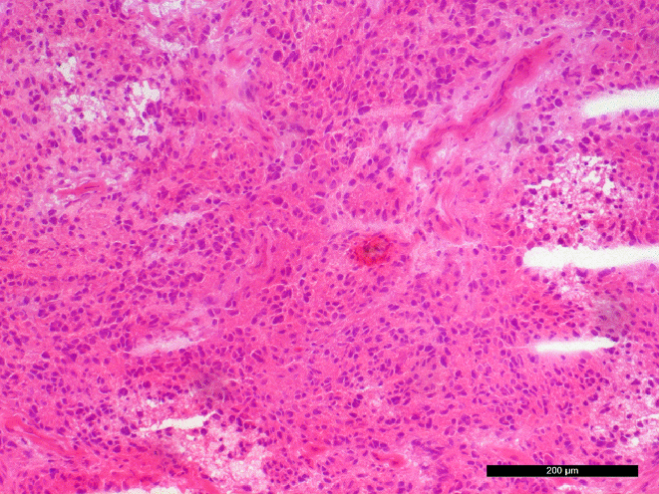
haematoxylin and eosin (H&E) stained cryostat section reveals a glial tumour with increased cellularity and prominent vascular structures. Scale bar: 200 µm

A biopsy of the tumour mass was obtained for intraoperative neuropathological examination. Hematoxylin and eosin (H&E) stained cryostat sections revealed cerebellar tissue along with areas of glial tumour (Fig. 4). The tumour exhibited increased cellularity, exceeding what is typical for a pilocytic astrocytoma. Additionally, prominent vascular structures were noted. Following surgical resection, the tumour tissue was fixed in formaldehyde and embedded in paraffin (FFPE) for further histopathological analysis.

**Fig. 5:**
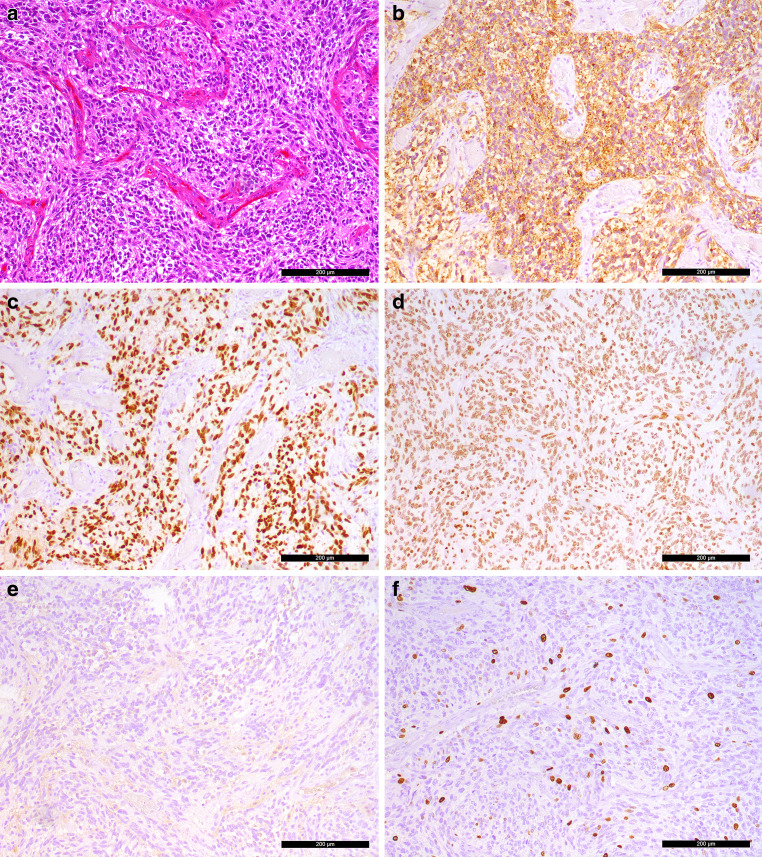
**a** H&E stained FFPE section confirms increased cellularity and vascular hypertrophy, with microvascular proliferation and increased mitotic activity. **b** Immunohistochemical reaction shows strong positivity for glial fibrillary acidic protein (GFAP, *brown*). **c** Tumour cells are positive for OLIG2 (*brown*). **d** Nuclear expression of ATRX is retained (*brown*). **e** IDH1-R132H immunohistochemistry is negative. **f** MIB‑1 proliferation marker indicates approximately 10% proliferation rate. Hematoxylin (*blue*) was used as counterstaining for immunohistochemical reactions. Scale bars: 200 µm

The H&E-stained FFPE section confirmed the intraoperative findings, showing a tumour with increased cellularity (Fig. 5a). Vascular hypertrophy was evident, including areas of microvascular proliferation. Increased mitotic activity was also observed, with two mitotic figures identified in 10 high-power fields (HPF). The tumour displayed a diffuse growth pattern into the surrounding cerebellar tissue, and occasional multinucleated giant tumour cells were detected.

Immunohistochemical analyses were performed to further characterize the tumour. Tumour cells demonstrated strong positivity for glial fibrillary acidic protein (GFAP) (Fig. 5b) and were also positive for OLIG2 (Fig. 5c). Nuclear expression of ATRX was retained (Fig. 5d), while IDH1-R132H immunohistochemistry was negative (Fig. 5e). The proliferation marker MIB‑1 indicated a proliferation rate of approximately 10% (Fig. 5f).

**Fig. 6:**
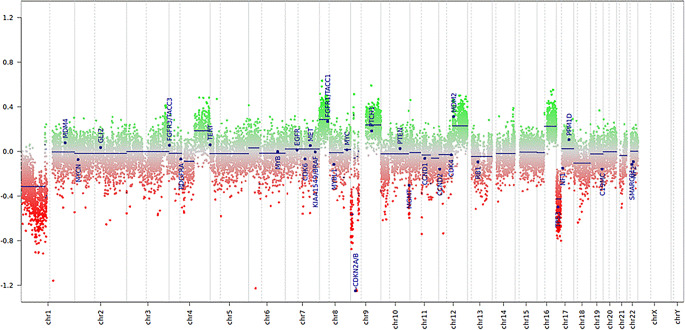
Copy number variation profile demonstrates loss of chromosome 1p, gain of 12q, and homozygous loss of CDKN2A/B as features commonly associated with this tumour type

Molecular analysis of the tumour was also conducted. Using multiplex ligation-dependent probe amplification (MLPA), a homozygous loss of CDKN2A and CDKN2B was detected. No signal was found for the mutation-specific probe for BRAF-V600E, indicating the presence of wild-type sequences. An 850k EPIC array was subsequently performed, and the copy number variation profile confirmed the loss of CDKN2A/B, along with 1p loss and gains in 12q, which are characteristic of high-grade astrocytomas with piloid features. Using the brain tumour classifier v12.8, the sample matched the methylation class of high-grade astrocytoma with piloid features, with a calibrated score of 0.96468 (Fig. 6).

## Diagnosis

### High-grade astrocytoma with piloid features (HGAP)

The fifth edition of the WHO classification of central nervous system tumours defines high-grade astrocytoma with piloid features (HGAP) as an astrocytic glioma with a distinct DNA methylation profile, often exhibiting glioblastoma-like histological features [30–33]. Genetic alterations in MAPK pathway genes are frequently observed, and these are often accompanied by homozygous deletions of the CDKN2A and/or CDKN2B loci, as well as ATRX mutations or loss of nuclear ATRX expression [25, 30]. In the current case, the criteria for HGAP are met, as we identified a distinct DNA methylation profile consistent with this entity, in addition to the homozygous deletion of CDKN2A/B.

In earlier versions of the WHO classification, this tumour type was discussed within the context of pilocytic astrocytomas, specifically those with anaplastic features [30]. However, HGAP is now recognized as a distinct tumour category due to its unique DNA methylation profile and aggressive clinical behaviour [30].

The patient has a documented history of pilocytic astrocytoma, first diagnosed in 2009, with histological evaluation conducted at an external institution. Although HGAP most commonly arises de novo, evidence suggests that it can also develop from pre-existing lower-grade astrocytic tumours, particularly pilocytic astrocytomas. While we were unable to evaluate the 2009 samples using the currently available 850k EPIC array technology, it is possible that the original tumour already exhibited some features of HGAP. Nevertheless, given the patient’s extended survival, it is more likely that the tumour had not yet transformed into HGAP in 2009 but has since progressed into this higher-grade entity.

Prognostic data for HGAP is primarily derived from a single retrospective study, which reported a 5-year overall survival rate of approximately 50% [25]. Patients with HGAP generally have shorter overall survival compared to those with conventional pilocytic astrocytomas (CNS WHO grade 1) and IDH-mutant astrocytomas (CNS WHO grade 3), but exhibit better survival outcomes than patients with IDH-wildtype glioblastomas [25, 30]. Notably, the clinical behaviour of HGAP aligns closely with tumours classified as CNS WHO grade 3 [25, 30].
